# The combination of Schisandrin C and Luteolin synergistically attenuates hepatitis B virus infection via repressing HBV replication and promoting cGAS-STING pathway activation in macrophages

**DOI:** 10.1186/s13020-024-00888-z

**Published:** 2024-03-18

**Authors:** Zhixin Wu, Xiaomei Zhao, Ruisheng Li, Xinru Wen, Ye Xiu, Minjuan Long, Junjie Li, Xiuqin Huang, Jincai Wen, Xu Dong, Yingjie Xu, Zhaofang Bai, Xiaoyan Zhan, Xiaohe Xiao

**Affiliations:** 1 School of Pharmacy, Hunan University of Traditional Chinese Medicine, Changsha, 410208 China; 2 Department of Hepatology, The Fifth Medical Center of Chinese PLA General Hospital, Beijing, 100039 China; 3 Research Institute of Department of Infectious Diseases, Fifth Medical Center of Chinese PLA General Hospital, Beijing, 100039 China; 4 National Key Laboratory of Kidney Diseases, Beijing, China

**Keywords:** Liuwei Wuling tablets, Schisandrin C, Luteolin, Combination therapy, Co-culture, HBV

## Abstract

**Background:**

HBV infection can result in severe liver diseases and is one of the primary causes of liver cell carcinoma-related mortality. Liuwei Wuling tablet (LWWL) is a traditional Chinese medicine formula, with a protecting liver and decreasing enzyme activity, usually used to treat chronic hepatitis B with NAs in clinic. However, its main active ingredients and mechanism of action have not been fully investigated. Hence, we aimed to screen the active ingredient and effective ingredient combinations from Liuwei Wuling tablet to explore the anti-herpatitis B virus activity and mechanism.

**Methods:**

Analysis and screening of effective antiviral components in LWWL by network pharmacology, luteolin (Lut) may be a compound with significant antiviral activity. The mechanism of antiviral action of Lut was also found by real-time PCR detection and western blotting. Meanwhile, we established a co-culture model to investigate the antiviral mechanism of Schisandrin C (SC), one of the main active components of Schisandra chinensis fructus (the sovereign drug of LWWL). Next, HBV-infected mice were established by tail vein injection of pAAV-HBV1.2 plasmid and administered continuously for 20 days. And their antiviral capacity was evaluated by checking serum levels of HBsAg, HBeAg, levels of HBV DNA, and liver levels of HBcAg.

**Results:**

In this study, we conducted network pharmacology analysis on LWWL, and through in vitro experimental validation and data analysis, we found that luteolin (Lut) possessed obviously anti-HBV activity, inhibiting HBV replication by downregulating hepatocyte nuclear factor 4α (HNF4α) via the ERK pathway. Additionally, we established a co-culture system and proved that SC promoted activation of cGAS-STINIG pathway and IFN-β production in THP-1 cells to inhibit HBV replication in HepG2.2.15 cells. Moreover, we found the combination of SC and Lut shows a greater effect in inhibiting HBV compared to SC or Lut alone in HBV-infected mice.

**Conclusion:**

Taken together, our study suggests that combination of SC and Lut may be potential candidate drug for the prevention and treatment of chronic hepatitis B.

**Supplementary Information:**

The online version contains supplementary material available at 10.1186/s13020-024-00888-z.

## Introduction

Hepatitis B virus (HBV) infection is a global public health concern [1] that can lead to chronic hepatitis B (CHB), elevating the risk of liver cirrhosis and hepatocellular carcinoma (HCC) [2]. Currently, interferon (IFN) and nucleoside/nucleotide analogues (NAs) are approved anti-HBV medications [3, 4], proven effective in suppressing HBV replication. While IFN can effectively block HBV replication and achieve viral clearance [5], its application is restricted due to administration methods and severe side effects [6, 7]. NAs, including entecavir, tenofovir disoproxil fumarate, tenofovir alafenamide fumarate and tenofovir amibufenamide [8], target HBV polymerase to suppress HBV replication [9] and are extensively utilized in clinical anti-HBV therapy. Nevertheless, a significant proportion of CHB patients necessitate long-term treatment of NAs, with a high rate of virological recurrence after discontinuation [10, 11], which represents a significant factor influencing treatment effectiveness.

The medical and scientific communities continue to explore the mechanisms of antiviral action against HBV and the diversity of drug targets. At present, there is an interest in the development of novel direct-acting antiviral drugs and Immune-modulating drugs. The main mechanism of action of direct-acting antiviral drugs is to reduce viral transcription and antigen levels by targeting the HBV life cycle and HBV gene expression [12]. The novel Immune-modulating drugs against HBV infection can enhance the immune response to HBV by stimulating innate immune reactions and adaptive immune responses [13].

The interaction between the antiviral immune response of the host cell and the immune evasion mechanisms of the hepatitis B virus (HBV) plays a pivotal role in the pathogenesis of HBV infection [7, 14]. Robust adaptive immune responses are capable of effectively controlling HBV infection during the acute phase, and immune activation is likewise linked to the suppression of HBV DNA and the spontaneous control of viral replication in chronic HBV disease [15]. Agonists of TLR3, RIG-I/MDA5, and STING have been reported to effectively suppress HBV replication in liver cells [16–18], suggesting that promoting the activation of innate antiviral pathways contributes in HBV clearance. Therefore, immunomodulatory drug has become a successful and promising treatment method for controlling and suppressing viral replication in patients with chronic hepatitis B [5], by enhancing antiviral immunity or suppressing liver inflammation.

Meanwhile, Traditional Chinese medicine (TCM), guided by a holistic perspective, has extensive experience and distinctive merits to cure hepatitis B, also play an essential part in regulating the body's state and improving self-immunity [19, 20]. Liuwei Wuling tablets (LWWL) are composed of schisandrae chinensis fructus (Wu Weizi or WWZ), ligustri lucidi fructus (Nv Zhenzi or NZZ), forsythiae fructus (Lian Qiao or LQ), curcumae rhizome (E Zhu or EZ), field sowthistle herb (Qu Maicai or QMC), and ganoderma spore powder (Lingzhi Baozifen or LZBZF). They can nourish the kidneys and liver, improve liver blood supply, reduce portal hypertension, and clear heat and toxins and have antiviral activity [21]. Moreover, clinical and experimental studies over the years have shown that LWWL exhibits remarkable anti-inflammatory and hepatoprotective effects, enhance immune function, and possess antiviral properties [22]. It can synergistically and effectively inhibit HBV virus replication, when used in combination with NAs or α-interferon to treat chronic hepatitis B [23–25]. Simultaneously, its application in the treatment of chronic hepatitis B has the potential to enhance the comprehensive clinical efficacy, including improvement of clinical symptoms, liver function indicators such as alanine aminotransferase (ALT), aspartate aminotransferase (AST), total bilirubin (TBIL) as well as a decline in levels of the hepatitis B virus. The study shows that LWWL has antiviral effect in vitro and in vivo [26], but the main active ingredients and mechanisms are not clear.

Luteolin (Lut) is a natural flavonoid compound [27] in ligustri lucidi fructus, forsythiae fructus, and field sowthistle herb in Liuwei Wuling tablets, exhibits notable anti-inflammatory, anti-neurodegenerative and anticancer activities. Furthermore, Luteolin demonstrates antiviral effects against various viral strains [28–30].

Schisandrin C (SC) is the primary and most abundant active ingredient isolated from Schisandrae chinensis fructus lignans [31], which has been reported that SC boosted the activation of cGAS-STING pathway [32]. Activating the cGAS-STING signaling pathway has been found to effectively suppress the replication of hepatitis B virus (HBV) in vivo and play a vital role in host’s antiviral response [33, 34]. Additionally, SC has been shown to attenuate HBV replication in vivo.

In our research, we assessed the therapeutic effect of the combination of LWWL and ETV in the treatment of hepatitis B. At the same time, we offer a new combination of compounds to treat HBV infection. Lut can directly suppress HBV replication by downregulating hepatocyte nuclear factor 4α (HNF4α) via the MAPK/ERK pathway, while SC inhibits HBV replication by enhancing the activation of the cGAS-STING pathway in immune cells. Meanwhile, we evaluated the combined therapeutic effect of SC and LUT in HBV-infected mice. The data showed the combination of Lut and SC has a more significant antiviral impact compared to the single drug. It suggests the combination of SC and Lut therapy for HBV may be a promising candidate for the treatment of hepatitis B.

## Materials and methods

### Reagents and antibodies

Schisandrin C (SC, HY-N0690), Luteolin (Lut, HY-N0162), Wogonin (Wog, HY-N0400), Kaempferol (Kae, HY-14590) and Quercetin (Que, HY-18085) were from MedChemExpress (New Jersey, NJ, United States). Acacetin (Aca, T3981) was form TargetMol (Boston, United States). Phorbol-12-myristate-13-acetate (PMA, tlrl-pma) was from Invitrogen (Carlsbad, CA, United States). Liuwei Wuling Tablet (LWWL) was from Shibo Jindu (Zibo, China). The antibodies were used for Western blot as follows: Rabbit monoclonal anti-pIRF3 (1:2000, ab76493) was purchased from Abcam; TMEM173/STING polyclonal antibody (1:2000, 19851-1-AP), IRF3 polyclonal antibody (1:2000, 11312-1-AP), HSP90 polyclonal antibody (1:5000, 13171-1-AP) were obtained from Proteintech (Chicago, IL, United States). Certified Fetal Bovine Serum (FBS, C04001) was from VivaCell (Shangha, China). DMEM (PYG0073) and RPMI 1640 (PYG0006) were purchased from BOSTER (Wuhan, China). StarFect II Transfection Reagent (C102) was obtained from GenStar (Beijing, China). Taq Pro Universal SYBR qPCR Master Mix (Q712-02) was from Vazyme (Nanjing, China).

### Cell line and co-culture

The steady HBV replication cell line HepG2.2.15 was used to evaluate the effects of luteolin, Schisandrin C and Liuwei Wuling Tablet in vitro. In addition to 10% fetal bovine serum and 1% penicillin–streptomycin, DMEM (Dulbecco's Modified Eagle Medium) was used to cultivate the HepG2.2.15 cells. While THP-1 cells were grown in RPMI 1640 medium with 10% fetal bovine serum and 1% penicillin–streptomycin as supplements. The THP-1 cells were treated with 100 nmol/L PMA (phorbol 12-myristate 13-acetate) for 4–5 h before to the studies in order to differentiate them into macrophages.

The indirect co-culture experiments were conducted using a 6-well transwell dish. The bottom well of the transwell dish containing DMEM serum medium. was seeded with HepG2.2.15 cells at a density of 2.5 × 10^5^ cells/ml. Meanwhile, the upper insert (0.4 μm pore size) containing 1640 serum medium, were seeded with HepG2.2.15 cells at a density of 1.2 × 10^6^ cells/ml. The cells were divided into 4 groups: normal, normal + SC, ISD, and ISD + SC. The normal group did not receive treatment. The normal + SC group was treated with 20 µM SC for 24 h at the bottom well. The ISD group was stimulated with ISD for 2 h. After that, we put transwell chamber which seeded with HepG2.2.2.15 cells on the top of 6-well transwell dish containing PMA-primed THP-1 cells. And the ISD + SC group was stimulated with ISD for 2 h after treated with SC for 2 h. After that, we put transwell chamber on the top of THP-1cells.

### Cell viability assay

In 96-well plates, HepG2.2.15 cells were seeded at 2 × 10^5^ cells/ml at 100 μl/well, respectively. After overnight incubation in a 37 ℃ incubator, the cells were given varied concentrations of luteolin or Liuwei Wuling Tablet at different. According to the manufacturer's recommendations, CellTiter-Glo luminescence assay kit (Promega, United States) was used to assess the cytotoxicity of luteolin, Liuwei Wuling Tablet in HepG2.2.15 cells.

### Animal model and treatment

The experimental subjects consisted of male C57BL/6J mice at the age of 7 weeks, which were purchased from SPF Biotechnology Co, Ltd. (Beijing, China). The mice were kept in a setting free of pathogens with unlimited access to food and water throughout the duration of the experiment. The animal experimental procedures followed the guidelines for the care and use of laboratory animals and were approved by the Animal Ethics Committee of the Fifth Medical Center of PLA General Hospital (Beijing, China).

Male C57BL/6J mice at 7 weeks of age were intravenously injected with 20 μg of pAAV-HBV1.2 plasmid (obtained from XJ Wang, Academy of Military Medical Sciences) or vehicle, corresponding to 10% of the mouse's body weight in saline, administered within 5–8 s. After establishing the mouse model for 3 days, blood samples were collected from the orbit to assess the levels of HBsAg, HBeAg, and HBV DNA in the serum using a commercial assay kit (WanTai BioPharm). Following that, to verify the effects of the combination of W and entecavir in vivo, the pAAV-HBV1.2-replicating mice were divided into 4 groups as follows: normal saline group, LWWL (2 g/kg) group, ETV (1 g/kg) group, and LWWL (2 g/kg) in combination with ETV (1 g/kg) group, for a total of 20 days. 6 mice were included in each group. Also, in order to assess the antiviral effects of the combination of SC and Lut, the mice injected with plasmids were divided into 4 groups with 6 mice each group, which were treated with saline, SC (20 mg/kg), Lut (20 mg/kg), and Lut (20 mg/kg) in combination with SC (20 mg/kg) group.

Serum samples were assessed for the presence of HBeAg, HBsAg, and HBV DNA. Immunohistochemistry was performed to detect HBcAg (anti-hepatitis B virus core antigen antibody [10E11], ab8639, Abcam) expression in the liver tissue of mice.

### Western blotting

We employed Western blot to assess the protein expression of p-IRF3, IRF3, STING, and p-ERK in cell lysates. HSP90 was used as a loading control. Protein extraction followed a standard procedure, where protein samples were heated to 105 ◦C and then dissolved in a 10% SDS-PAGE solution. Based on their molecular weight, the proteins were sorted by electrophoresis and then wet transferred onto a polyvinylidene fluoride (PVDF) membrane. Subsequently, the samples were supplemented with 5% skimmed milk and incubated for 1 h. Following this, the samples were exposed to primary antibodies overnight at 4 °C. After three washes with TBST, secondary antibodies were applied, followed by an additional three TBST washes. Finally, protein expression levels were determined using commercial ECL kits and enhanced chemiluminescence assay membranes, following the manufacturer's instructions.

### Enzyme-linked immunosorbent assay (ELISA)

Both the HBsAg and HBeAg ELISA kits were obtained from Wantai. Human IFN-beta bioluminescent ELISA kits 2.0 was purchased from Invivogen. The ELISA was assessed based on the manufacturer’s guidelines.

### Quantitative real-time PCR (qRT-PCR)

Cell lysis was conducted using TRizol reagent (BSC51M1) followed by purification of total RNA as per the manufacturer's instructions. Subsequently, cDNA synthesis was performed using StarScript III All-in-one RT Mix with gDNA Remover (GenStar, A230-10). qRT-PCR was carried out using 2 × RealStar Fast SYBR qPCR Mix (Low ROX). The relative expression levels of the target genes were determined using the ΔΔCT method on a Quant Studio 6 Real-Time PCR instrument (Applied Biosystems). The primer sequences utilized in this study are presented in Table 1, with actin serving as the reference gene for normalization.

**Table 1:** Quantitative PCR primer sequences

Target gene name	Gene sequence (5′–3′)	Source
Human actin	CATGTACGTTGCTATCCAGGC	From PMID: 33142842
	CTCCTTAATGTCACGCACGAT	
Human IFN-β	TCCAAATTGCTCTCCTGTTG	From PMID: 35577759
	GCAGTATTCAAGCCTCCCAT	
HNF4α	CCATCAGAAGGCACCAACC	From PMID: 26656210
	TCTTTGTCCACCACGCACT	

### Network pharmacology analysis

Through the utilization of TCMSP (http://tcmspw.com/), the active chemical compounds present in Wu Weizi, E Zhu, Nv Zhenzi, and Lian Qiao were screened based on their oral bioavailability (OB) ≥ 30% and druglikeness (DL) ≥ 0.18. Additional key chemical ingredients were incorporated by referencing relevant literature.

HBV-related targets were acquired from 5 databases using the keyword "hepatitis virus B" as follows: DisGeNET (https://www.disgenet.org/), GeneCards (https://www.genecards.org/), Online Mendelian Inheritance in Man (OMIM, https://omim.org/), Therapeutic Target Database (TTD, http://db.idrblab.net/ttd/), Drugbank (https://www.drugbank.ca/). These databases were utilized to search for all target genes. Subsequently, Uniprot was employed to rectify the gene names of the aforementioned targets. A network depicting the interaction between active components of LWWL and HBV-related targets was established based on their interaction data. The visualization of this network was achieved using Cytoscape software (version 3.9.1).

STRING (https://string-db.org/) is an interactive gene database search tool that provides protein–protein interaction (PPI) information. The common targets were entered into STRING11.0, and Homo Sapiens was limited Organism, minimum required interaction score was set to the high confidence (0.400). Cytoscpace3.9.1 was applied to generate the PPI network diagram of the common targets, with adjustments made to node size and color based on degree in the diagram.

### GO analysis and KEGG pathway enrichment analysis

Combining GO and KEGG enrichment analysis can obtain comprehensive functional information about a large number of genes from a macro perspective and identify drug-disease signaling pathways. The common targets identified after STRING processing were uploaded to the Metascape database for GO and KEGG analysis, yielding relevant data on cellular component (CC), molecular function (MF), biological process (BP), and KEGG pathways. The top 10 GO terms and top 20 KEGG pathways with the smallest *P* values were chosen and imported into the bioinformatics mapping website (http://www.bioinformatics.com.cn/) to create bar graphs and bubble graphs.

### Statistical analyses

Statistical analysis was performed using GraphPad Prism V8.0 program. Multiple comparisons were conducted using one-way ANOVA with Dunnett's post-hoc test, while differences between two groups were assessed using unpaired Student's *t*-test. The results of every experiment were presented as mean ± SD. A significance level of *P* < 0.05 was considered statistically significant.

## Results

### LWWL significantly inhibits HBV replication

**Fig. 1:**
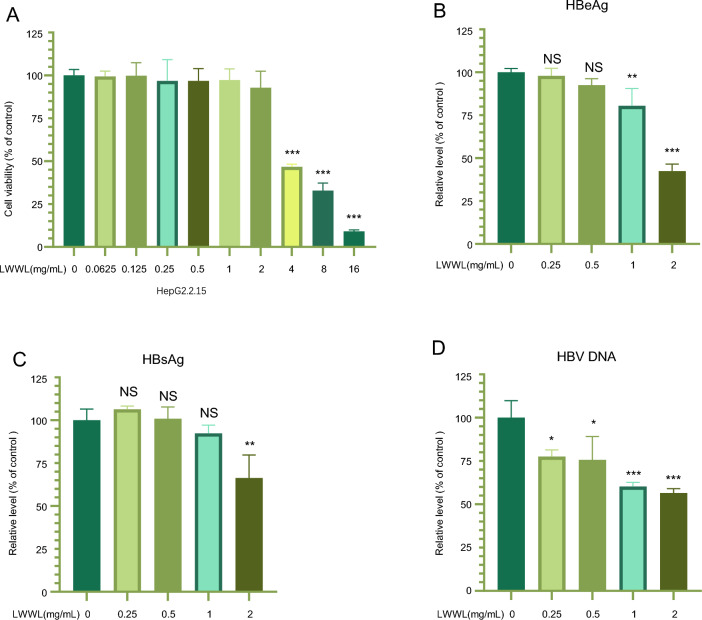
LWWL inhibited the replication of HBV in vitro. HepG2.2.15 cells were treated with the indicated concentrations of LWWL for 24 h. Cell death was measured by CellTiter-Glo luminescent assay (**A**). HepG2.2.15 cells were treated with the indicated concentrations of LWWL for 24 h. Then the supernatant was collected to detect the levels of HBeAg, HBsAg, HBV DNA (**B**–**D**). The data are presented as mean ± SEM from triplicates, *P < 0.05, **P < 0.01, ***P < 0.001, NS: not significant (one-way ANOVA with Dunnett’s post-hoc test). HepG2.2.15 cells were subjected to treatment with varying concentrations of LWWL for a duration of 24 h. Cell death was assessed using the CellTiter-Glo luminescent assay (**A**). HepG2.2.15 cells were exposed to the respective concentrations of LWWL for 24 h, and the supernatant was collected for analysis of HBeAg, HBsAg, and HBV DNA levels (**B**–**D**). The data presented are mean values ± standard error of the mean (SEM) from triplicates, *P < 0.05, **P < 0.01, ***P < 0.001, and NS denotes not significant (one-way ANOVA with Dunnett’s post-hoc test)

To determine a noncytotoxic concentration suitable for the anti-HBV study, we assessed the cytotoxic effects of LWWL on HepG2.2.15 cells. Our findings revealed that when the concentration of LWWL was below 2 mg/ml, the rate of cell death remained below 5% (Fig. 1a). This indicates that LWWL at a concentration of 2 mg/ml or lower exhibited minimal cytotoxicity towards the cells. Consequently, a maximum concentration of 2 mg/ml of LWWL was employed in this study. After being treated with LWWL for 24 h, the levels of HBsAg and HBeAg in the culture media were then assessed using ELISA. The results demonstrated that LWWL effectively decreased HepG2.2.15 cell production of HBsAg, HBeAg, and HBV DNA. Furthermore, the inhibition exhibited a dose-dependent relationship (Fig. 1b–d). To sum up, these results support LWWL’s anti-HBV activity in vitro.

**Fig. 2:**
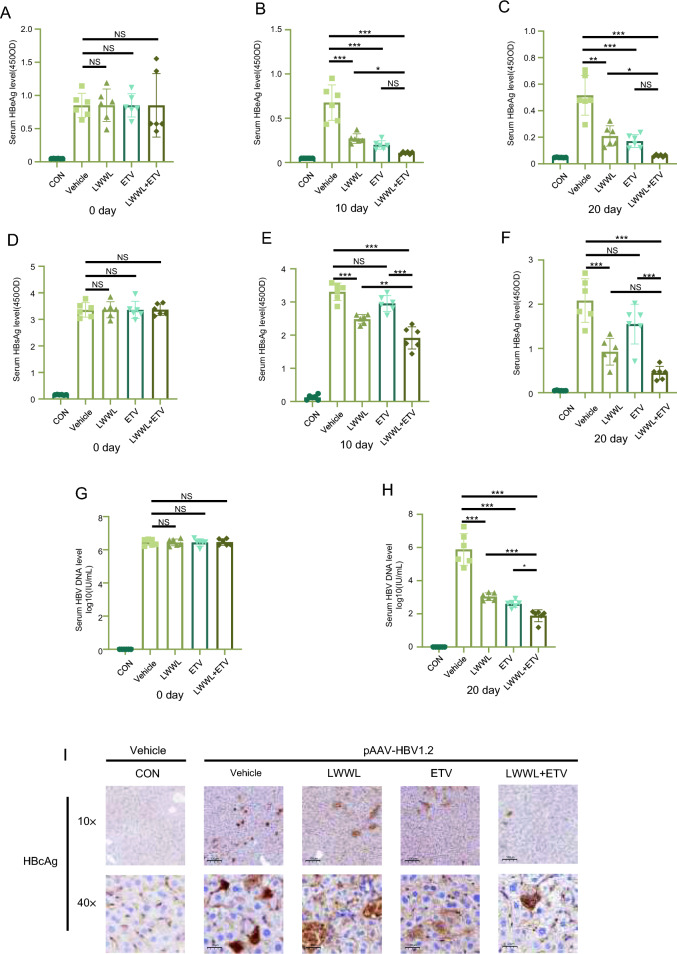
The combination of LWWL and ETV reduces hepatitis B virus replication in vivo. C57BL/6J mice were hydrodynamic injection of pAAV/HBV1.2 plasmids at a dose of 20 μg through the tail vein within a duration of 6–8 s, controls were injected with saline equivalent to 10% of the mouse’s body weight. After 3 days, blood was taken from the orbit to detect the levels of HBsAg, HBeAg and HBVDNA in the serum. Upon observing an increase in the expression of HBsAg, HBeAg, and HBV DNA in the mice, the mice injected with plasmids were divided into four groups (n = 6). These groups received treatment with saline, LWWL, ETV, and a combination of LWWL and ETV, respectively, at concentrations below the minimal liver-cytotoxic level for a period of 20 days. The control group was administered saline. The levels of serum HBeAg (**A**–**C**), HBsAg (**D**–**F**), and HBV DNA (**G**, **H**) were measured at specified time points. At the end of the 20-day period, the mice were sacrificed, and the expression of HBcAg in the liver was assessed using immunohistochemistry (**D**). The scale bars represent 100 μm (top row) and 20 μm (bottom row). Data are shown as mean ± SEM, *P < 0.05, **P < 0.01, ***P < 0.001, NS: not significant (one-way ANOVA with Dunnett’s post-hoc test)

Building on this foundation, we have further confirmed whether the combination of LWWL and ETV is better than the single use, we tested a series of viral marker on hepatitis B virus, such as HBsAg, HBeAg and so on. The results showed a significant decrease in the levels of both HBeAg and HBV DNA in the serum of mice treated with LWWL, ETV or their combination (Fig. 2a–f). Furthermore, the level of HBsAg in the serum of mice treated with LWWL also decreased (Fig. 2g, h). Compared to the monotherapy group, the combination group showed a better decrease in the levels of HBeAg and HBV DNA in the serum. It was indicated that the synergistic inhibitory effect of the combination of LWWL and ETV on hepatitis B virus was significantly superior to that of treating with LWWL or ETV alone. Consistent with the immunohistochemical assessment, the combination treatment was more effective in reducing HBcAg in tissues compared to individual treatment as expected (Fig. 2i).

The results demonstrated that compared to the single use, the combination of LWWL and ETV had a greater antiviral effect in vivo.

### Network pharmacology-based analysis

In order to investigate the main components of LWWL for the treatment of hepatitis B, we conducted a network pharmacology analysis. Within the TCMSP database, a total of 36 active compounds of LWWL were identified based on the criteria of OB ≥ 30 and DL ≥ 0.18. Additionally, the main compounds of Qu Maicai and Lingzhi Baozifen were supplemented based on relevant literature. Consequently, we obtained a total of 50 active ingredients of LWWL (detailed compound information is provided in Additional file 1: Tables S1 and S2). The active ingredients of LWWL were further matched to potential targets, resulting in the inclusion of a total of 250 targets.

Through the utilization of the following 5 databases, namely DisGeNET, GeneCards, OMIM, TTD, and Drugbank, we identified 1449, 474, 593, 20, and 39 confirmed or potential HBV targets, respectively. To ensure consistency, all targets were standardized using Uniprot. Duplicate targets were subsequently removed, resulting in a final set of 2000 targets.

**Fig. 3:**
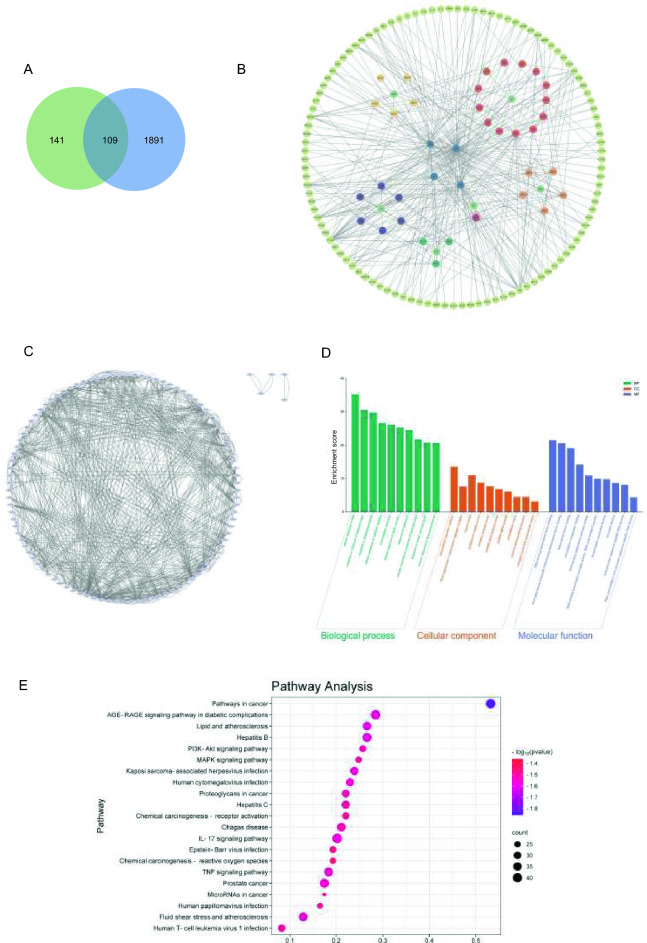
Network pharmacological analysis of LWWL on hepatitis B virus treatment. **A** Venn diagram of LWWL and HBV therapeutic targets. **B** The LWWL-Ingredients-Target-HBV network. **C** Protein–protein interaction network of common targets of LWWL and HBV. **D** GO enrichment analysis. **E** KEGG enrichment analysis

The intersection of active ingredients and disease targets was visualized using a Venn diagram (Fig. 3a), which revealed 109 common targets. As a result, we discovered that the 37 active compounds were associated with 109 HBV-related targets. To visualize the LWWL active ingredient-target-disease network, we utilized Cytoscape 3.9.1. The network consisted of 152 nodes and 350 edges (Fig. 3b). Analysis of the network demonstrated a centrality value of 0.55 and a heterogeneity value of 1.89, illustrating the synergistic therapeutic effect of LWWL through the interaction of multiple ingredients with multiple targets. Detailed information on the top 10 compounds is provided in Table 2. Also, 109 intersection targets in the figure were input into STRING11.0, and got 102 targets and made them a PPI network diagram of LWWL for HBV by Cytoscape (Fig. 3c).

**Table 2:**
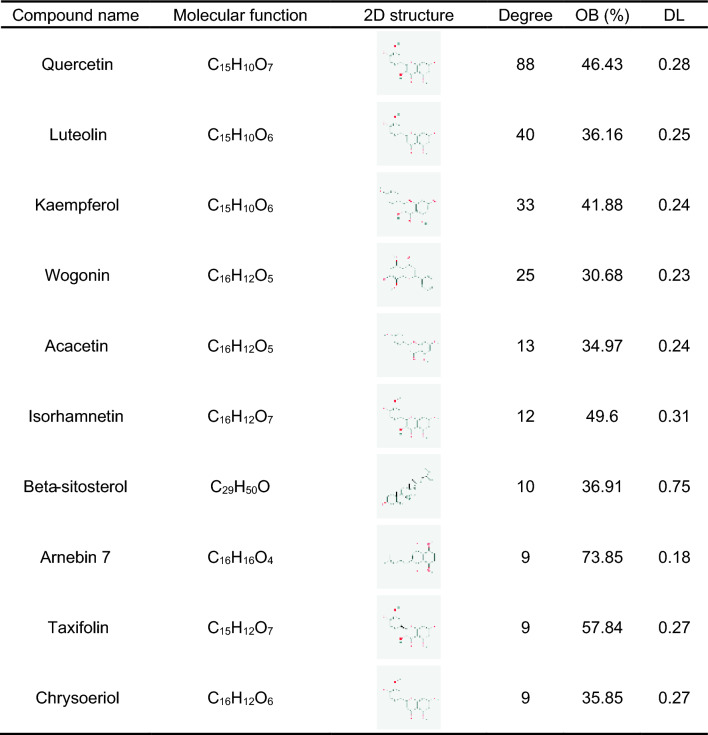
Compounds in the ingredient-target-disease network of Liuwei Wuling Tablets (top 10)

Metascape was utilized to conduct GO analysis of targets in PPI network. It shows that the numbers of cellular components (CC), molecular functions (MF), and biological processes (BP) are 65, 125, 15,900 respectively (*P* < 0.05, the bar graph of the top 10 is shown in Fig. 3d). Following KEGG Pathway analysis (*P* < 0.05), we identified a total of 180 signaling pathways. Top 6 signaling pathways are: Pathways in cancer, AGE-RAGE signaling pathway in diabetic complications, Lipid and atherosclerosis, Hepatitis B, PI3K-Akt signaling pathway, MAPK signaling pathway, (the bubble chart of the top 20 is shown in Fig. 3e).

### Lut inhibits HBV replication in HepG2.2.15 cells via ERK-mediated downregulation of HNF4a

**Fig. 4:**
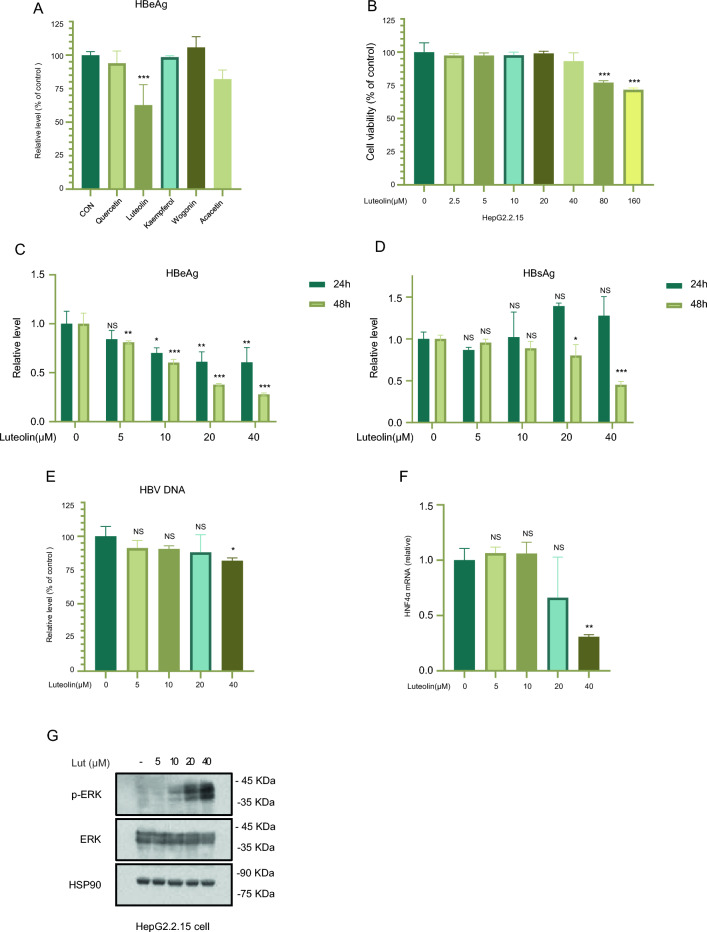
Lut inhibits the expression of HNF4α and HBV replication through activating MAPK/ERK pathway. HepG2.2.15 cells were treated with indicated compounds for 24 h. Then the supernatant was collected to detect the levels of HBeAg (**A**). HepG2.2.15 cells were treated with the indicated concentrations of Lut for 48 h. Cell death was measured by CellTiter-Glo luminescent assay (**B**). HepG2.2.15 cells were treated with the indicated concentrations of Lut for 24 or 48 h. then the supernatant was collected for the determination of HBeAg, HBsAg, HBV DNA (**C**–**E**). HepG2.2.15 cells were treated with Lut (5, 10, 20 and 40 μM) for 6 h, the mRNA was acquired for the measurement of HNF4α (**F**) by qRT-PCR. HepG2.2.15 cells were incubated with Lut (5, 10, 20 and 40 μM) for 24 h, and the blot intensity of p-ERK1/2 (**G**) was analyzed through Western blot. The data are presented as mean ± SEM from triplicates, *P < 0.05, **P < 0.01, ***P < 0.001, NS: not significant (one-way ANOVA with Dunnett’s post-hoc test)

In order to investigate the antiviral effects of compounds in LWWL, the top five drugs were chosen based on network pharmacology degrees, and their effects on the concentrations of HBeAg in the HepG2.2.15 cells’ supernatant were examined by using ELISA. The data demonstrated a significant reduction in the level of HBeAg by luteolin (Fig. 4a). It suggests that luteolin (Lut) may have antiviral effect in vitro.

For further study of evaluating the antiviral ability of Lut in vitro, we initially assessed the cytotoxicity of Lut in HepG2.2.15 cells using the CellTiter-Glo luminescent assay. Following a 48-h exposure of HepG2.2.15 cells to Lut, the cell viability was measured, and the results indicated that concentrations of Lut below 40 µM resulted in a cell death rate of less than 5%, suggesting that Lut at a concentration of 40 µM or lower exhibited minimal cytotoxicity (Fig. 4b). Therefore, Lut with a maximum concentration of 40 µM was used. Subsequently, the impact of Lut on HBV replication in HepG2.2.15 cells was examined. After treating Lut for 24 and 48 h, the levels of HBsAg and HBeAg in the culture media were assessed. The results revealed that Lut inhibited the release of HBsAg and HBeAg from HepG2.2.15 cells (Fig. 4c, d). The inhibitory effects of Lut on HBsAg and HBeAg were dose-dependent and found to increase with the concentration of luteolin used, indicating a clear dose-dependent manner, also enhanced with time in HepG2.2.15 cells. Also, the level of HBV DNA was evaluated, it showed the inhibition of HBV DNA replication was significant within the concentration range from 10 to 40 μM of luteolin (Fig. 4e).

Hepatocyte nuclear factor 4α (HNF4α) is a member of the HNF family, plays a key role in HBV transcription, particularly in the generation of pgRNA [35, 36]. The activation of the MAPK signaling pathway has the potential to disturb the communication between enhancers and promoters of HNF4α, resulting in the down-regulation of HNF4α expression [37]. It has been reported before that Lut could activate MAPK/ERK pathway to downregulate the expression of HNF4α [30], we have confirmed Lut significantly increase the phosphorylation of ERK (p-ERK) exhibited a dose-dependent manner (Fig. 4f, g), and inhibited the expression of HNF 4 α in a dose-dependent manner.

Overall, the findings revealed that Lut significantly inhibited HBV replication via ERK-mediated downregulation of HNF4α.

### SC promotes the activation of cGAS-STING pathway and interferon production in THP-1 cells to suppress HBV replication in HepG2.2.15 cells

In addition to direct antiviral drugs, there is a growing interest in the role of immunity which consist of adaptive and innate immunity in the treatment of HBV. cGAS-STING pathway, one of the important antiviral pathways [38, 39], has been reported to play an essential role in inhibiting HBV. SC, one of the main lignan of Schisandra chinensis (sovereign drug of LWWL), has been reported to facilitate the activation of cGAS-STING pathway in macrophages [32].

We proposed that SC augmented the activation of the cGAS-STING pathway in macrophages to induce interferon secretion, thereby suppressing the replication of HBV DNA in hepatocytes and achieving an antiviral effect.

**Fig. 5:**
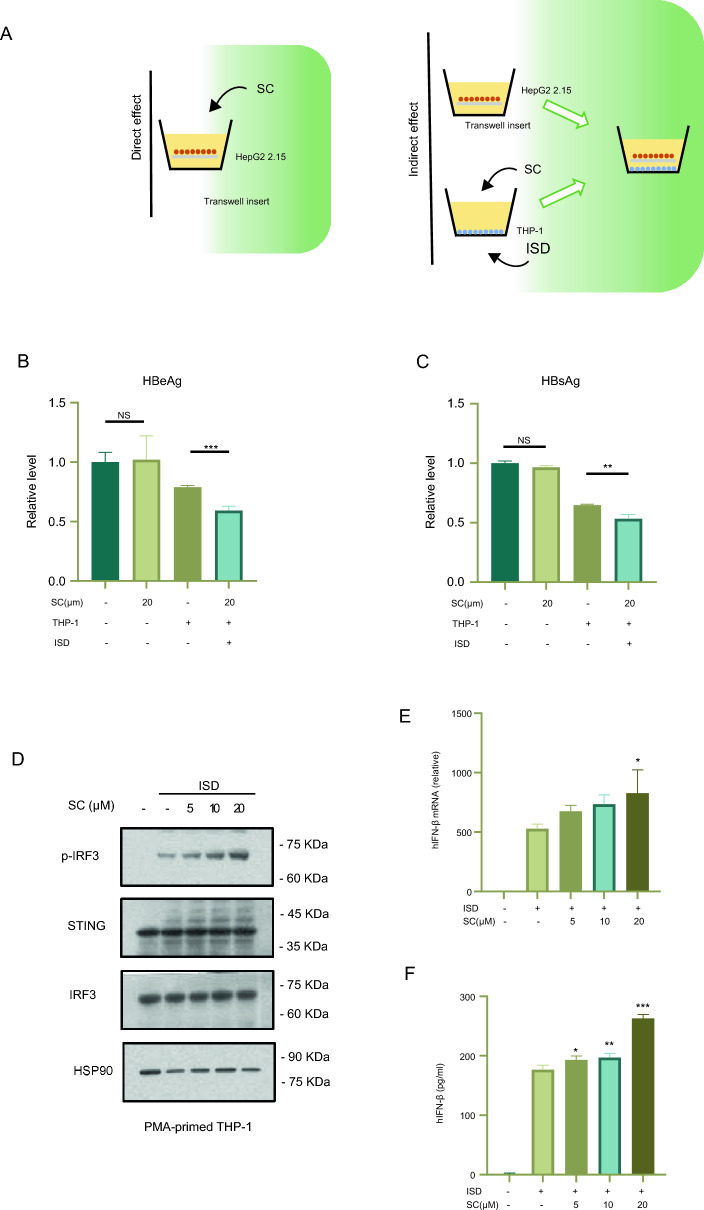
SC has an antiviral effect in vitro by increasing the production of IFN via ISD-induced cGAS-STING pathway activation. PMA-primed THP-1 cells which were seeded in the 6-well plate were treated with SC (20 µM) for 2 h, then stimulated with ISD for 2 h. Put Transwell chamber which seeded with HepG2.2.2.15 cells on the top of 6-well transwell dish which seeded with THP-1 cells (**A**). After 24 h we collected the supernatant to measure the levels of HBeAg and HBsAg (**B**, **C**). PMA-primed THP-1 cells were incubated with SC (5, 10, and 20 μM) for 2 h, then stimulated with ISD for 2 h and the blot intensity of p-IRF3, STING, IRF3 (**D**) was analyzed through Western blot. PMA-primed THP-1 cells were treated with DMSO or SC for 2 h, then stimulated with ISD for 3 h, induction of IFN β mRNA was measured by qRT-PCR (**E**). PMA-primed THP-1 cells were treated with DMSO or SC for 2 h, then stimulated with ISD for 6 h, secreted IFN β were detected by ELISA (E). Data are shown as mean ± SEM from triplicates, **P < 0.01, ***P < 0.001, NS: not significant (one-way ANOVA with Dunnett’s post-hoc test or unpaired Student’s *t*-test)

To verify this hypothesis, we established a co-culture system of THP-1 and HepG2.2.15 cells. We put transwell chamber which seeded with HepG2.2.2.15 cells on the top of 6-well transwell dish containing THP-1 cells pretreated with SC or vehicle followed by ISD (Interferon Stimulatory DNA) transfection or not. After 24 h we collected the supernatant to measure the levels of HBsAg and HBeAg. The datas showed that in the context of ISD transfection, the HBeAg and HBsAg’s level of the co-culture group were obviously reduced, and there was a better reduction in the group given with SC (Fig. 5b, c). Also, we evaluated whether SC has a direct antiviral effect on HepG2.2.15 cells. The results indicated that treating HepG2.2.15 cells with SC did not lead to a decrease in the levels of HBsAg and HBeAg.

Next, we confirmed the effect of SC on the cGAS-STING signaling pathway, the results showed that the phosphorylation of IRF3 (p-IRF3) exhibited a dose-dependent increase (Fig. 5c). And the mRNA expression of interferon and the expression of IFN-β in the supernatant of THP-1 cells were evaluated. As anticipated, the expression of mRNA and interferon in the supernatant increased with the rise in concentration (Fig. 5d, e).

Above all, the results could suggest that SC can raise cGAS-STING pathway’s activation, resulting in the secretion of IFN-β and thereby decreasing the levels of HBsAg and HBeAg in the supernatant.

### A combination of SC and Lut significantly inhibits HBV DNA replication in vivo

**Fig. 6:**
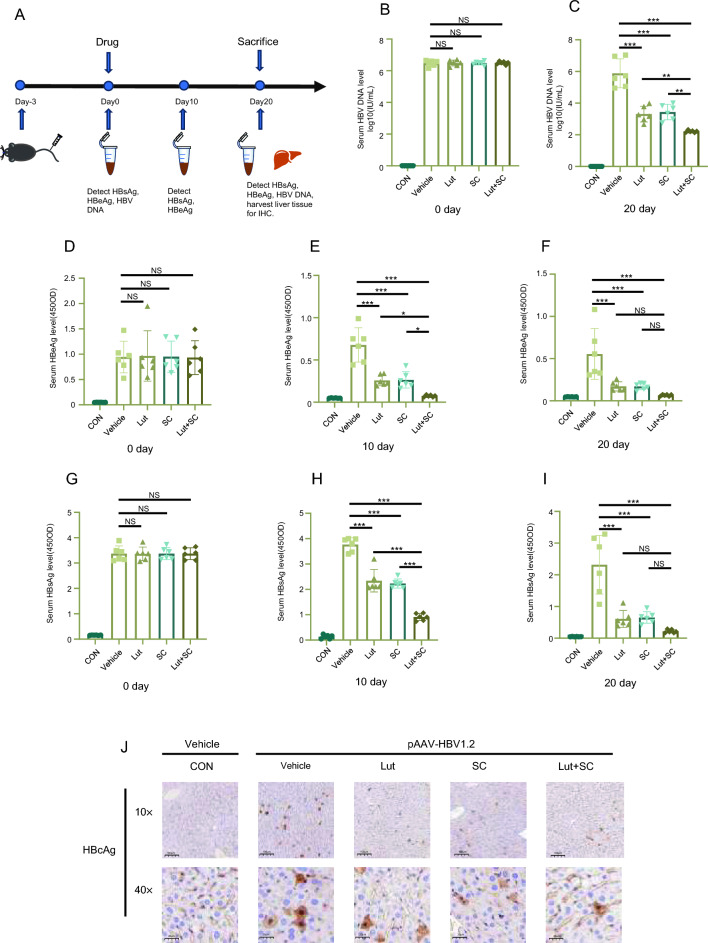
The combination of SC and Lut reduces hepatitis B virus replication in vivo. C57BL/6J mice were hydrodynamic injection with 20 μg of pAAV/HBV1.2 plasmids through the tail vein within 6 − 8 s, controls were injected with saline equivalent to 10% of the mouse’s body weight. After 3 days, blood was taken from the orbit to detect the level of HBsAg, HBeAg and HBVDNA in the serum. When the expression of HBsAg, HBeAg, and HBV DNA in mice increases, the mice injected with plasmids were divided into 4 groups (n = 6), which were treated with saline, Lut, SC, and their combination under the minimal liver-cytotoxic concentration within 20 days, respectively (**A**). The control group was given saline. Serum HBeAg (**D**–**F**), HBsAg (**G**–**I**) and HBV DNA (**B**, **C**) levels were measured for indicated times. Mice were sacrificed at the 20 day, the expression of HBcAg in liver were detected by immunohistochemistry (**J**), scale bars: 250 μm (top row); 50 μm (bottom row). Data are shown as mean ± SEM, *P < 0.05, **P < 0.01, ***P < 0.001, NS: not significant (one-way ANOVA with Dunnett’s post-hoc test)

Previous experiments have indicated that luteolin can suppress HBV replication by downregulating HNF4α through the MAPK/ERK pathway, while SC can directly promote innate immunity and enhance the activation of the cGAS-STING antiviral pathway to inhibit viral replication. Subsequently, we proceeded to assess whether the synergistic effect of SC and Lut demonstrated a superior anti-HBV activity when compared to their individual treatments in the HBV-infected mouse model. The PAAV/HBV1.2 plasmids was used to induce hepatitis B (Fig. 6a). After the injection of plasmid into the tail vein of mice, the level of HBsAg, HBeAg in the serum increased (Fig. 6d, g), indicating that the mice had been infected with hepatitis B virus, this was further confirmed by HBV DNA (Fig. 6b).

After that, the mice were randomly divided into 4 groups, except for the control group, and received gavages with saline (model group), SC (20 mg/kg), Lut (20 mg/kg), a combination of SC (20 mg/kg) and Lut (20 mg/kg) within 20 days. The data showed SC, Lut, and their combined treatment groups showed good effects in preventing hepatic B, and Inhibiting HBV replication, which the decrease in the levels of HBsAg and HBeAg in the serum can prove. Also, the combined group also showed better inhibition of HBsAg and HBeAg in the serum to the single drug group (Fig. 6e, f, h, i). Moreover, the coordinate repression of SC and Lut on reduction of HBV DNA in the serum was significantly superior than SC or Lut alone (Fig. 6c). HBcAg is an endonuclear protein of the hepatitis B virus, which plays an important role in the replication and assembly of the hepatitis B virus, and its test can be used for the diagnosis of hepatitis B, disease monitoring and prognosis assessment. Consistent with the results of liver histopathological evaluation, the combination therapy of SC and Lut also reduced the content of HBcAg in liver tissue more effectively than SC and Lut alone (Fig. 6j).

Thus, the combination of SC with Lut offered synergistic effects against the chronic hepatitis B.

## Discussion

In the study, we found that Lut in LWWL may have a potential antiviral effect against hepatitis B by network pharmacology, and confirmed it through cellular experiments. Moreover, SC was found to enhance the CGAS-STING pathway and activate immune cells' antiviral immune response to suppress viral replication. In further in vivo experiments, we discovered that the combination of SC and LUT exhibited a superior anti-hepatitis B effect, providing a promising therapeutic candidate for hepatitis B treatment.

LWWL, as a drug used clinically in combination with NAs to treat hepatitis B, has hepatoprotective and enzyme-lowering effects, as well as antiviral effects in vitro and in vivo [26, 40]. Building on this foundation, we further confirmed the antiviral effect of LWWL in hepatitis B by observing reductions in the level of HBV DNA, HBsAg, and HBeAg in serum.

Furthermore, we found that the combination of LWWL and entecavir exhibited a superior antiviral effect in HBV-infected mice model, suggesting a beneficial treatment outcome when LWWL is used in combination with ETV in clinical practice, thereby providing a therapeutic strategy for hepatitis B treatment.

Network pharmacology is an interdisciplinary field that integrates systems biology and network informatics to investigate the interactions and balance within biological networks [41, 42]. It provides a comprehensive approach to studying the effects of multi-component drugs on the human body at a systemic level. Through utilizing network pharmacology, researchers can analyze the effects of drugs on multiple targets and pathways, aiding in the identification of therapeutic targets for active drug ingredients. This approach has the potential to enhance drug efficacy and minimize adverse side effects.

In order to better clarify the main components and mechanisms of LWWL in the treatment of hepatitis B, we used network pharmacology to analyse them.

We established compound-disease-target network to identify the top five scoring compounds and performed in vitro experiments. The data showed Lut, which many traditional Chinese medicines in LWWL contain, had a superior antiviral activity in vitro. Through KEGG analysis, it was concluded that LWWL may treat hepatitis B by affecting the MAPK pathway. The data revealed that Lut can downregulate the expression of HNF4α by promoting MAPK/ERK pathway, to achieve antiviral effects, which is consistent with the previous study. On this basis, we also identified some other components through network pharmacology analysis, and further research is needed to determine whether these components have good anti-hepatitis B effects.

Subsequently, the principal active components and mechanisms of action of LWWL against HBV were analyzed through network pharmacology. We analyzed compound-disease-target network to identify the top five scoring compounds and performed in vitro experiments. The data showed Lut had a superior antiviral activity in vitro. Further experiments revealed that Lut inhibits the replication of HBV DNA by downregulating the expression of HNF4α.

Previous research has shown SC could enhance the activation of the cGAS-STING pathway and was able to inhibit HBV DNA replication in HBV-infected mice. However, it is not clear whether SC actually inhibits viral replication by activating antiviral immune response in macrophages. Therefore, we established a co-culture system of HepG2.2.15 and THP-1 cells to prove it in this experiment. In the co-culture system, SC can enhance the activation of the cGAS-STING pathway through the stimulation of ISD, leading to increased interferon production and decreased the secretion of HBsAg and HBeAg in the supernatant of HepG2.2.15 cells.

The combination therapy has the characteristics of regulating multiple components, multiple pathways, and multiple target points, which can produce a synergistic effect, bringing new opportunities for the development of traditional Chinese medicine. Combination therapy as a new intervention measure may be necessary tool for clinical management of HBV, which can involve inhibiting HBV viral replication and enhancing the immune system to strengthen HBV clearance.

Furthermore, we assessed whether the combined therapeutic effect of SC and Lut is superior to monotherapy in mice induced HBV infection. The study demonstrated that the combination of SC and Lut was more effective in reducing the levels of HBsAg, HBeAg, and inhibiting HBV DNA replication in the serum. Moreover, the decrease in HBcAg observed in immunohistochemistry confirmed that the combination of SC and Lut exhibited superior antiviral effects compared to monotherapy.

Due to the current treatment status of CHB, the goal of treatment has been increasingly focused on clinical cure in recent years [41]. is defined as continuous absence of HBsAg and persistent undetectable HBV DNA with or without production of hepatitis B surface antibody (HBsAb), while cccDNA and integrated HBV DNA may persist in infected liver cells [43]. We found that the combination of SC and Lut significantly reduced the level of HBsAg in the serum, suggesting that combination therapy may increase the chance of achieving a functional cure for hepatitis B.

Our research provides potential candidate drugs for the treatment of chronic hepatitis B through combination therapy by targeting different pathway and highlights the broad prospects of combination therapy in the treatment of chronic hepatitis B.

### Supplementary Information

**Additional file 1. Table S1**: Active ingredients of Liuwei Wuling tablets. **Table S2**: Targets in protein–protein interaction network of common targets of LWWL and HBV.

## Data Availability

The data produced from this study are available from the first author and the corresponding author on reasonable request.
