# Supplementary material for: The combination of Schisandrin C and Luteolin synergistically attenuates hepatitis B virus infection via repressing HBV replication and promoting cGAS-STING pathway activation in macrophages
Source: Chin Med. 2024 Mar 18;19:48. doi: 10.1186/s13020-024-00888-z (PMC10946137; doi:10.1186/s13020-024-00888-z)

Table S1. **Active ingredients of Liuwei Wuling Tablets.**


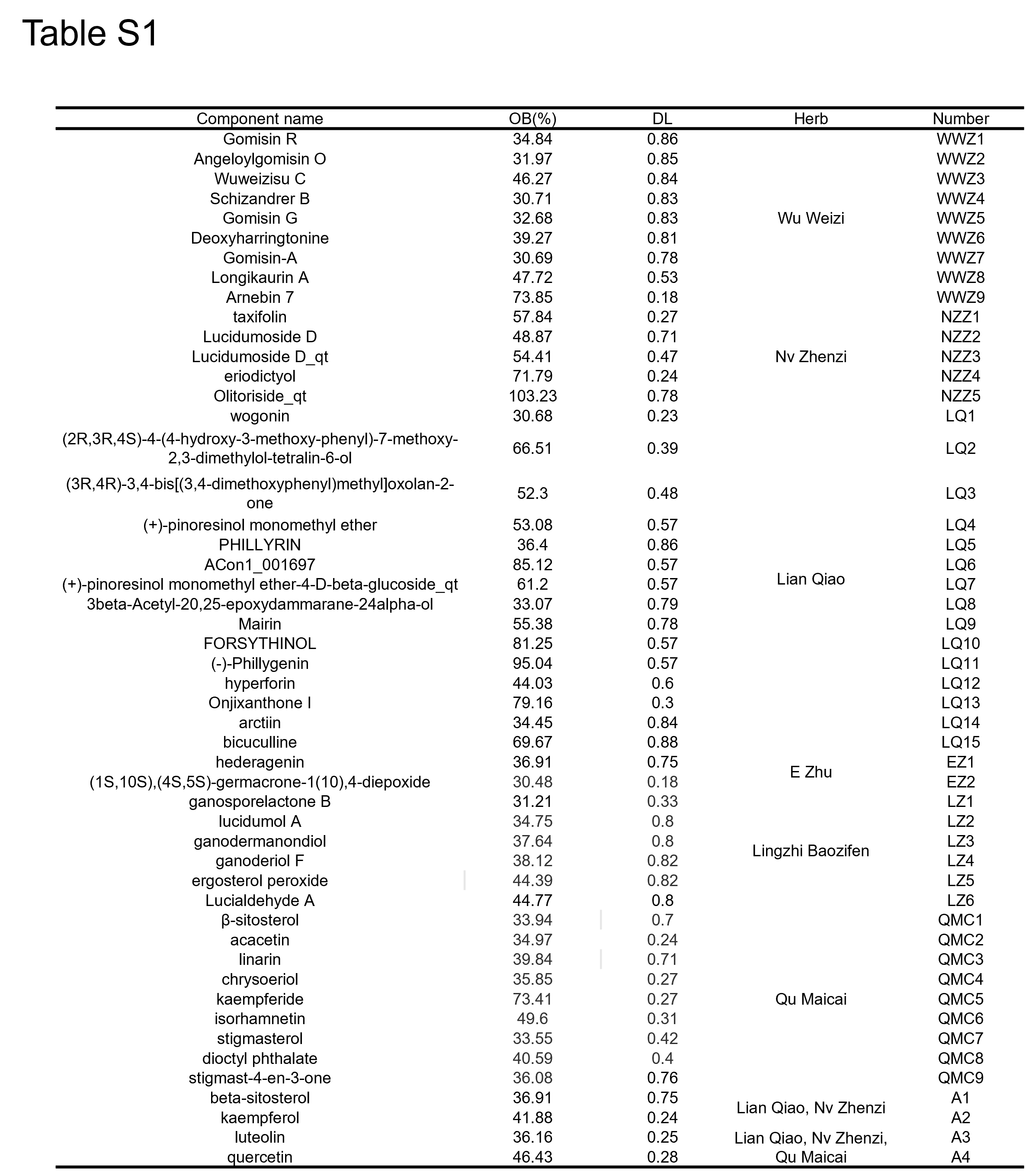


Table S2. **Targets in protein-protein interaction network of common targets of LWWL and HBV.**


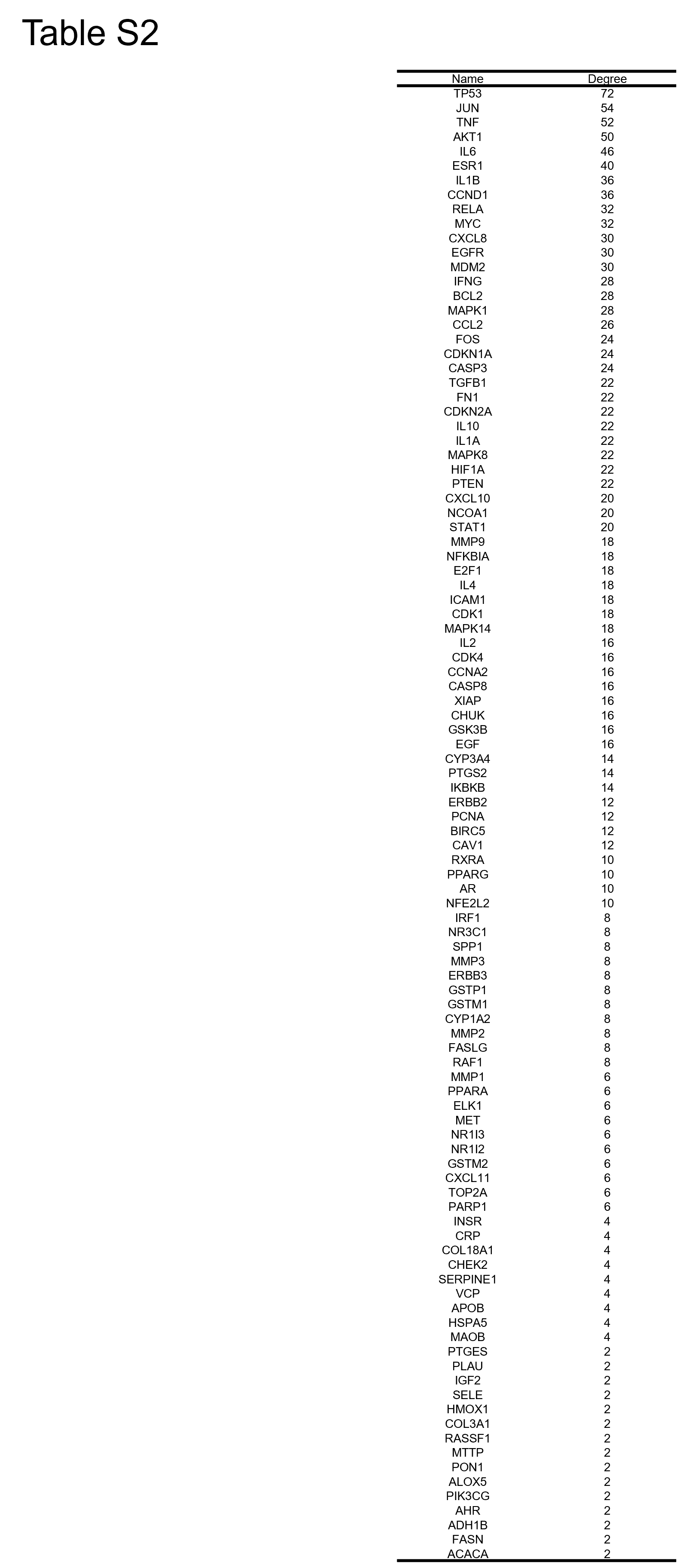

Supplement: Supplementary file 1 — Additional file 1. Table S1: Active ingredients of Liuwei Wuling tablets. Table S2: Targets in protein–protein interaction network of common targets of LWWL and HBV. [file 13020_2024_888_MOESM1_ESM.docx]
